# Long-term safety and efficacy of fecal microbiota transplantation in 74 children: A single-center retrospective study

**DOI:** 10.3389/fped.2022.964154

**Published:** 2022-10-11

**Authors:** Biao Zou, Sheng-Xuan Liu, Xue-Song Li, Jia-Yi He, Chen Dong, Meng-Ling Ruan, Lei Xu, Tao Bai, Zhi-Hua Huang, Sai-Nan Shu

**Affiliations:** ^1^Pediatric Department, Tongji Hospital, Tongji Medical College, Huazhong University of Science and Technology, Wuhan, China; ^2^Division of Gastroenterology, Union Hospital, Tongji Medical College, Huazhong University of Science and Technology, Wuhan, China

**Keywords:** fecal microbiota transplantation, children, adverse event, efficacy, safety, follow-up

## Abstract

**Background:**

Fecal microbiota transplantation (FMT) is an effective treatment for intestinal and extra-intestinal disorders. Nonetheless, long-term safety and efficacy remain major challenges for FMT applications. To date, few long-term follow-up studies have been published on FMT in children.

**Methods:**

Retrospective reviewed the medical charts of 74 patients who underwent 508 FMT courses between August 2014 and July 2019 at our medical center. All the FMT procedures followed uniform standards. Baseline characteristics pre-FMT and follow-up data were collected at 1, 3, 6, 12, 36, 60, and 84 months after FMT. All potential influencing factors for adverse events (AEs) were analyzed and assessed using regression analyses.

**Results:**

A total of 70 (13.7%) short-term AEs occurred in twenty-six patients (35.1%). Most AEs (88.5%) occurred within 2 days post-FMT. A total of 91.4% of the AEs were self-limiting. Ulcerative colitis (UC) and within four times of FMT were associated with a higher rate of AEs (*p* = 0.028 and *p* = 0.021, respectively). The primary clinical remission rate after FMT was as high as 72.9%. Twenty-five children were followed for more than 5 years after FMT. The clinical remission rates gradually decreased over time after FMT. During follow-up, none of the patients developed autoimmune, metabolic, or rheumatologic disorders or tumor-related diseases. However, nine children developed rhinitis, five developed rhinitis, were underweight, and six developed constipation.

**Conclusions:**

FMT is a safe and effective treatment for dysbiosis in children. The long-term efficacy of FMT for each disease decreased over time. Moreover, multiple FMTs are recommended 3 months post-FMT for recurrent diseases.

## Introduction

The human gut contains approximately 1,000–1,200 bacterial species in symbiosis with their host. This complex community of microorganisms is important for the development and maturation of the digestive system, competitive exclusion of pathogens, and immune system (1, 2). Dysbiosis is associated with a wide range of diseases, including gastrointestinal and non-digestive conditions.

Fecal microbiota transplantation (FMT) is a process in which a presumed healthy and diverse microbiome is transplanted to a patient using a nasogastric tube, colonoscopy, or enema, to remodel the intestinal flora balance (3, 4). By 2015, approximately 15,000 FMTs had been carried out worldwide. By the end of March 2022, there were more than 360 clinical trials on FMT, with more than 50 in children under 17. FMT clinical trials have also been conducted for the treatment of Crohn's disease (3), irritable bowel syndrome (5), autism (6), ulcerative colitis (3), obesity (7), acute graft-vs.-host disease (8), and constipation (9), showing great promise for diverse conditions beyond *Clostridiodies difficile* infections (10).

Although FMT benefits patients, several concerns remain to be addressed, including the spread of unwanted pathogens, short-term adverse events (AEs), and long-term safety and efficacy (11). While there are some studies on the safety and efficacy of FMT in adults (12–14), there are few studies in children and even fewer long-term follow-up data on FMT in children. The safety and efficacy of FMT in children have become a barrier to its further implementation. The pediatric gastroenterology department at the Tongji Hospital of Tongji Medical College, Huazhong University of Science and Technology, is one of the earliest and largest centers to carry out FMT in children in China. Using the FMT method, We have a lot of successful cases in our team (15, 16). In our study, we reviewed the medical charts of 74 children who received 508 FMT and ten patients were followed up for seven years after FMT. We also reviewed the long-term safety and efficacy of FMT in children.

## Methods

### Ethics

This study was approved by the Medical Ethics Committee of Tongji Hospital, Tongji Medical College, Huazhong University of Science and Technology (TJ-C20220312). Written informed consent for FMT treatment was obtained from the parents or legal guardians of all pediatric subjects.

### Study population

We retrospectively reviewed the medical charts of 74 pediatric patients who received FMT as part of their treatment between August 2014 and July 2019 at the Department of Pediatrics at Tongji Hospital of Tongji Medical College, Huazhong University of Science and Technology. These patients included children diagnosed with refractory *C. difficile* infection (CDI), recurrent infantile allergic colitis (AC), refractory chronic intractable diarrhea (CID), ulcerative colitis (UC), recurrent atopic dermatitis (AD), and refractory functional constipation (FC).

Patients met the following criteria in the study: (i) no alteration in medicines for at least 1 week before FMT; (ii) refractory CDI, defined as at least two episodes of *C. difficile* infection confirmed by stool *C. difficile* toxin A/B; a diagnosis of UC based on clinical, radiologic, endoscopic, and histologic evidence; refractory FC according to the Rome III or Rome IV criteria was constipation that persisted for more than 6 months of standard treatment; refractory CID was based on diarrhea lasting more than 2 months after routine treatment; recurrent AC was based on rectal bleeding after hypoallergenic milk powder for more than 1 month; a diagnosis of recurrent AD based on eczema with itch after hypoallergenic milk powder, skin care, and pharmacotherapy for more than 1 month; and (iii) no treatment-related contraindications for FMT. Patients who were followed up for less than 3 months were excluded.

All subjects were examined at baseline (before FMT) and 1, 3, 6, 12, 36, 60, and 84 months after the FMT procedure. Subjects were assessed at each point of follow-up, they completed relevant laboratory tests (e.g., blood routine, biochemistry, c-reaction protein, erythrocyte sedimentation rate, and fecal routine examinations), re-evaluated the improvement of clinical symptoms and monitored height and weight, etc.

### The donor screening

A total of 74 healthy donors were recruited, with one recipient to one donor in the study. Eligible donors, including children of the same age (e.g., relatives, trusted friends, and unrelated) or their mothers, were recruited according to the following criteria (17, 18): (i) no history of infectious diseases (e.g., tuberculosis); (ii) no history of metabolic diseases (e.g., obesity and diabetes); (iii) no gastrointestinal diseases or functional disorders, including chronic fatigue, inflammatory bowel disease, and irritable bowel syndrome; (iv) no allergic diseases (e.g., eczema); (v) no antibiotic taken in the past 3 months; (vi) no autoimmune diseases; (vii) no drug abuse history; (viii) on a regular diet during donation of the material.

The subjects and donors underwent rigorous serological and stool tests within seven days before FMT donation. Clinical tests included complete blood count, chemistry, hepatitis virus, HIV, and common enteric pathogens (Supplementary Table 1). They had to rescreen and reevaluate any abnormalities in their symptoms and signs. Fecal 16S RNA or macrogene sequencing was performed if necessary.

### FMT procedure

FMT is initiated at the time of diagnosis or disease flares. Concomitant medications were continued when the FMT was administered. The number of FMT infusions was grouped into single FMT (one day) or multiple FMTs (2–18 days continuously). No bowel preparation (cleanup) was performed before FMT. Donor feces were collected 1–2 h pre-FMT, attenuated, and mixed with sterile normal saline (1 mg feces was attenuated with 5 mL saline). Samples were filtered through sterile gauze, and a 100 mL fecal suspension was prepared for FMT. The fecal suspension was poured into a sterile cup for FMT procedure within 1 h. The routes of administration included colonoscopy and enema. Generally, the first FMT procedure is performed during colonoscopies. If the fecal microbiota sample was obtained from a bacteria bank at −80°C, it was thawed at 37 °C and FMT was performed within 1 h. After infusion, the subjects were asked to hold a fixed position (>25°semi-reclining) for more than 4 h. The FMT procedure followed a uniform standard for each patient. FMT was administered at different frequencies according to disease type and severity.

### Data collection

We reviewed a database of 74 patients with FMT. The data used in this study were extracted from the medical records at our center. Baseline demographic data were collected for all patients, including demographic characteristics, diagnosis, relationship between donors and recipients, FMT procedure, number of FMT infusions, FMT clinical efficacy, short-term AEs, and long-term clinical efficacy and safety. To evaluate clinical efficacy and safety, laboratory data were collected pre-FMT and at 1, 3, 6, 12, 36, 60, and 84 months post-FMT, including ESR, CRP, and Pediatric Ulcerative Colitis Activity Index (PUCAI). Height, weight, and stool frequency (no/week) were also collected.

### Safety and efficacy assessment

The diagnostic criteria and curative effect evaluation of the different diseases in the present study are shown in Table 1. Clinical response was defined as the sum of clinical remission and improvement. Children with weight <3rd percentile were diagnosed as underweight according to the standardized growth curve of height and weight of children and adolescents aged 0–18 in China (26). The first assessments were performed in the inpatient department within 1 week after completion of FMT, and subsequent assessments of the efficacy and safety of FMT were performed at different follow-up time points. If the subjects required additional medications at any evaluation point in time, they were considered clinically invalid. Accordingly, any increase in medication use was considered clinically unresponsive.

**Table 1:** The diagnostic criteria and curative effect evaluation of different diseases in patients enrolled in this study.

**Classification of diseases**	**Diagnose criteria**	**Clinical remission**	**Clinical improvement**
FC (19, 20)	Rome III Diagnostic Criteria for Functional Gastrointestinal Disorders. *J Gastrointestin Liver Dis*. (2006) 15:307–312. The New Rome IV Criteria fo Functional; Gastrointestinal Disorders in Infants and Toddlers. *Pediatr Gastroenterol Hepatol Nutr*. (2017) 20:1–13.	Spontaneous defecation ≥ 3 times per week	The clinical symptoms did not reach clinical remission, but improved significantly compared with before treatment
UC (21, 22)	Pediatric inflammatory bowel disease. *World J Gastroenterol*. (2006) 12:3204–3212. Inflammatory Bowel Disease in Children and Adolescents. *JAMA Pediatr*. (2015) 169:1053–1060.	PUCAI <10 points	PCDAI decreased by more than 10 points and PUCAI <30
CID (23)	Clinical approach and management of chronic diarrhea. *Acta Med Indones*. (2013) 45:157–165.	The number of defecations as well as the amount is normal	The number of defecation is reduced to 2~3 times/day
AC (15)	Fecal microbiota transplantation induces remission of infantile allergic colitis through gut microbiota re-establishment. *World J Gastroenterol*. (2017) 23:8570–81.	The blood stool returned to normal	Blood in the stool and diarrhea improved but were not normal
CDI (24)	Guidelines for diagnosis, treatment, and prevention of *Clostridiodies difficile* infections. *Am J Gastroenterol*. (2013) 108:478–99.	The clinical symptoms of diarrhea and hematochezia were completely normal	The clinical symptom for CDI better than before, but not up to the standard of cure
AD (25)	Japanese Guideline for Atopic Dermatitis 2014. *Allergol Int*. (2014) 63:377–98.	SCORAD index ≤5 and SCORAD decreased by more than 30 points	SCORAD index decreased by more than 10 points

FC, functional constipation; UC, ulcerative colitis; CID, chronic intractable diarrhea; AC, infantile allergic colitis; CDI, C.difficile infection; AD, atopic dermatitis.

AEs were defined as any unwanted medical events emerging in the subjects after FMT was performed. An SAE was described as any AE leading to any of the following consequences: death, life-threatening experience, extended inpatient hospitalization, persistent or severe disability, or an important medical event (27). Causality between AEs and FMT was classified as either related or unrelated (27). AEs occurring within 1 month were determined to be short-term; after 1 month, long-term (28). AEs occurring within 1 month post-FMT were recorded daily. AEs occurring beyond 1 month were recorded during outpatient follow-up and monitoring. AEs were assessed based on the clinical symptoms, signs, and laboratory parameters. The potential long-term AEs under our monitor included infection due to unidentified pathogens, metabolic diseases, and growth constraints.

### Data analyses

Count data are presented as the number of cases (%), and descriptive statistics are presented as means (standard deviations) or medians (ranges). Statistical analyses were performed using the IBM SPSS Statistics version 25. The potential factors of FMT-related AEs in the 74 enrolled patients were analyzed using univariable and multivariable logistic regression analyses. A *p* < 0.05 was considered statistically significant in a two -side test. GraphPad Prism version 8 was used to generate graphics.

## Results

### Patients' demographic and clinical characteristics

**Figure 1:**
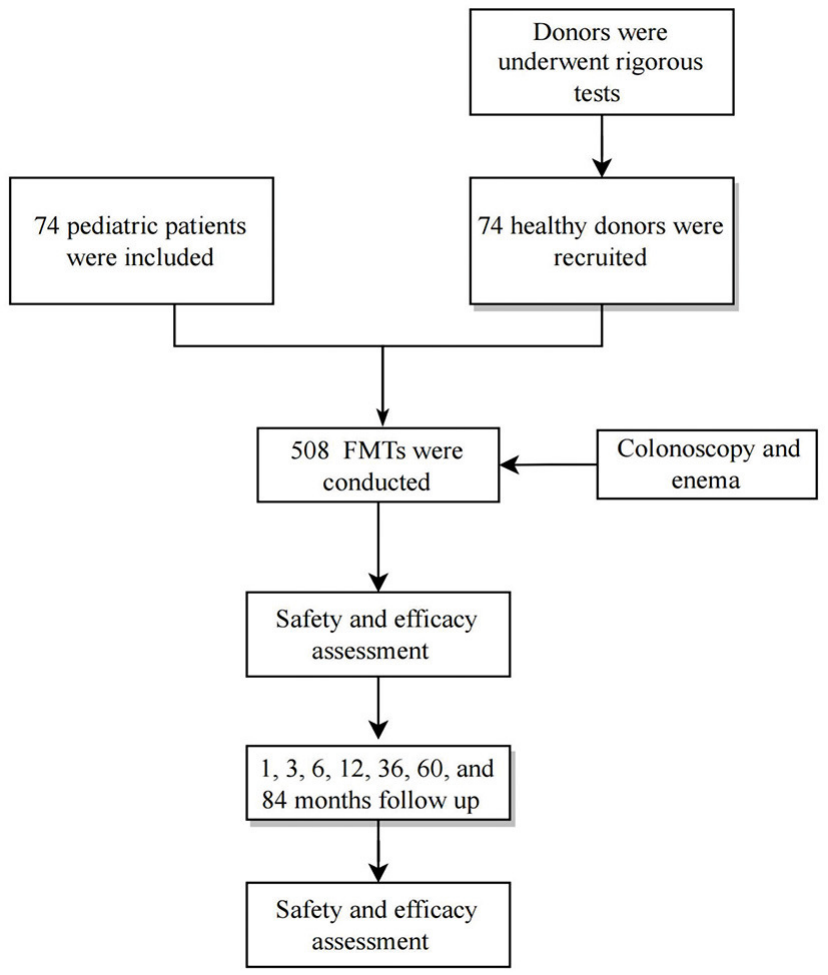
Flowchart of patient inclusion. FMT, fecal microbiota transplantation.

Seventy-four patients who underwent 508 FMT treatments between August 2014 and July 2019 were included (Figure 1). Sixty-eight patients who received two or more FMTs and had six disease types were included. Only six patients received a single FMT. All patients were routinely treated for >2 months before FMT. The mean patient age was 51.1 mo (54.3) (range, 4–168 months), 47 (63.5%) patients were male, and all the first FMT procedures were performed during hospitalization. Data on patient characteristics, disease classification, clinical efficacy, and AEs are shown in Table 2. Follow-up data were recorded from the beginning of the FMT treatment to March 2022.

**Table 2:** Clinical information for 74 patients.

	**Sum of patients (n)**	**male *n* (%)**	**Age [mean ±SD (range), mo]**	**Disease Duration (mo)**	**Treatment(s) before FMT**	**Concomitant medications**	**Clinical remission**	**Clinical improvement**	**Clinical invalid**	**AE(number of patients) *n* (%)**	**Total of FMT courses**	**Number of FMT-related AEs *n* (%)**	**Routes of administration**
CDI	7	7 (100.0)	56.5 ± 45.0 (10–113)	2	Vancomycin, Metronidazole	/	6 (85.7)	1 (14.3)	0	2 (28.6)	28	4 (14.2)	Enema+ Colonoscopy
CID	9	7 (77.7)	41.1 ± 46.3 (9–152)	2	Probiotics (Bifidobacteria), ORS, Zinc, Antibiotics	Probiotics (Bifidobacteria), ORS	6 (66.6)	1 (11.1)	2 (22.2)	4 (44.4)	37	5 (13.5)	Enema
FC	18	6 (33.3)	83.5 ± 41.9 (18–168)	6	Lactulose, Probiotics	Lactulose, Probiotics	12 (66.6)	3 (16.6)	3 (16.6)	5 (27.7)	172	11 (6.4)	Enema+ Colonoscopy
UC	12	10 (83.3)	100.8 ± 51.4 (56–163)	3	Mesalamine, prednisone	Mesalamine	8 (66.6)	2 (16.6)	2 (16.6)	8 (66.6)	181	43 (23.8)	Enema+ Colonoscopy
AD	6	4 (66.7)	23.8 + 25.3 (7–71)	3	Moisturizer, Amino acid formula	Amino acid formula	2 (33.3)	2 (33.3)	2 (33.3)	2 (33.3)	29	2 (6.9)	Enema
AC	22	13 (59.1%)	6.8 ± 2.3 (4–12)	2	Amino acid formula, Probiotics	Amino acid formula	20 (90.9)	2 (9.1)	0	5 (22.7)	61	5 (8.1)	Enema

CDI, C. difficile infection; AC, infantile allergic colitis; CID, chronic intractable diarrhea; UC, ulcerative colitis; AD, atopic dermatitis; FC, functional constipation; FMT, fecal microbiota transplantation; PEN, Partial enteral nutrition; ORS, oral rehydration salts.

### FMT efficacy

The FMT was relatively effective. The overall clinical response rate after FMT was 87.8%. Among them, 54 (72.9%) children had “clinical remission”, 11 (14.9%) had “clinical improvement, and nine (12.2%) had” invalid clinical symptoms. The best therapeutic effects were detected for AC (90.9%) and CDI (85.7%), whereas AD (33.3%) presented the poorest therapeutic effects. At the same time, the sleep of most children significantly improved after FMT.

### FMT safety

**Figure 2:**
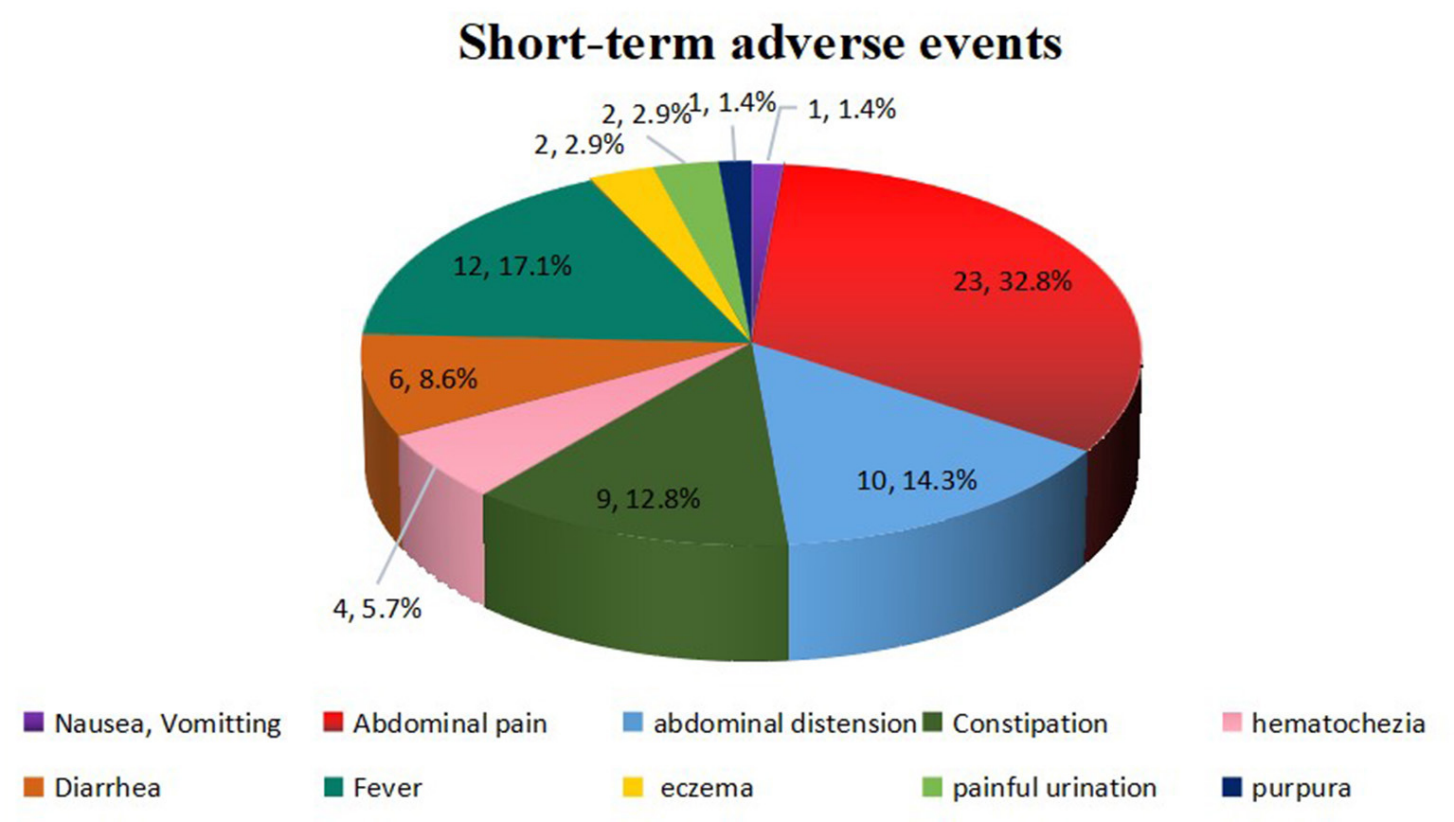
FMT-related short-term AEs. Overall, 70 AEs were observed in 508 FMT courses. These AEs included abdominal pain (*n* = 23), fever (*n* = 12), abdominal distension (*n* = 10), constipation (*n* = 9), diarrhea (*n* = 6), hematochezia (*n* = 4), eczema (*n* = 2), painful urination (*n* = 2), nausea or vomiting (*n* = 1), and purpura (*n* = 1). FMT, fecal microbiota transplantation; AEs, adverse events.

**Figure 3:**
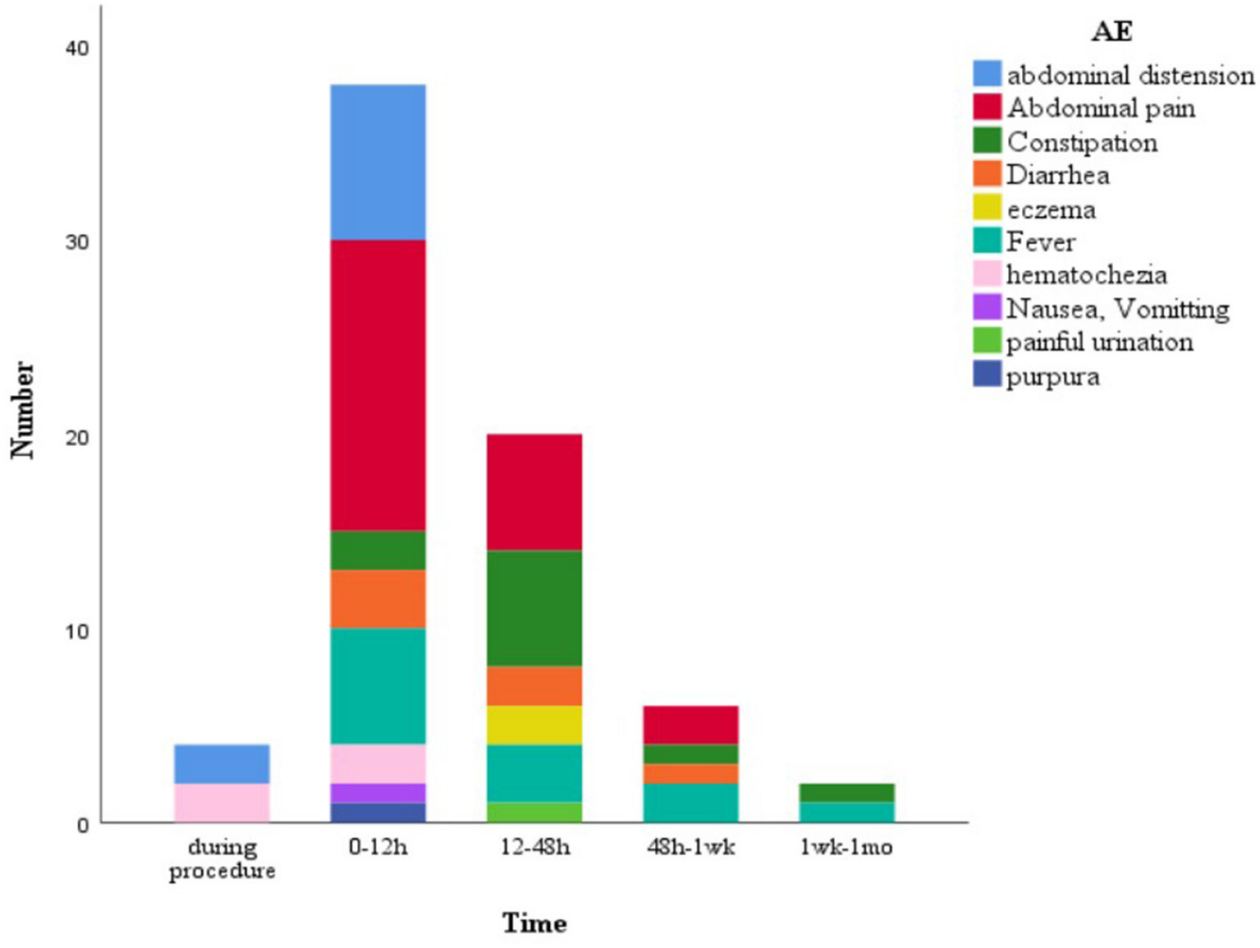
FMT-related short-term AEs at diferent time points. 62 AEs (88.5%) appeared within 48 h post-FMT. FMT, fecal microbiota transplantation; AEs, adverse events.

Overall, FMT was relatively safe. A total of 70 (13.7%) FMT-related short-term AEs occurred in 26 patients (35.1%), including four patients with four SAEs (Figure 2). Most AEs (88.5%) appeared within 48 h post-FMT (Figure 3). The most common AEs were abdominal pain (32.8%), fever (17.1%), and distension (14.3%). Moreover, most AEs were transient and self-limited (91.4%). The incidence of SAEs was only 0.79% (4/508), which could be improved using conventional drug interventions. When these patients underwent FMT again, there was still some occasional discomfort, but they could tolerate it.

### SAEs

SAEs were found in four courses of FMT (0.79%) in four patients and improved after conventional drug interventions. A child with AC developed vomiting, diarrhea, watery stools, and fever 3 days after FMT. Rotavirus infection was diagnosed and improved after symptomatic supportive treatment, which was suspected to be caused by donor stool infection. A child with CID developed pain and urgency in urination and fever 1 day after FMT and was finally diagnosed with a urinary tract infection. The symptoms improved 3 days after antibiotic application. The infection was considered to be caused by fecal bacterial infection of the urinary tract during the FMT procedure. Two children with UC developed fever 1 day after FMT. Routine blood tests and CRP were elevated, and inflammatory markers and body temperature returned to normal 3 days after antibiotic administration. These symptoms might be related to poor intestinal mucosal barriers and intestinal environments in children with UC and systemic infection with fecal bacteria through intestinal mucosal destruction after FMT.

### Risk factors related to FMT AEs

AEs were assessed based on the clinical symptoms, signs, and laboratory parameters. The univariate logistic regression analysis factors, including age (*p* = 0.032), disease type (*p* = 0.0018), number of FMT infusions (*p* = 0.0005), and administration routes (*p* = 0.036) had remarkable effects on AE occurrence (Table 3). Multivariable logistic regression analyses showed that the AE rate of AEs in the UC group than in the non-UC group (29.0 vs. 66.6%, *p* = 0.028). The incidence of FMT-related AEs in children with more than four FMTs was significantly lower than that in children with less than four FMTs (18.1 vs. 60.0%, *p* = 0.021).

**Table 3:** Potential factors influencing FMT-related AEs occurrence.

**Item FMT procedures**	**Subgroup *n* = 74**	**Number of AEs % (n/N)**	**Univariate *p* value**	**Multivariate**		
				**OR**	**95% CI**	***p***
Age group (n)	0–36 mo >36 mo	30.7 (12/39) 40.0 (14/35)	0.032	0.836	0.121–2.564	0.526
Disease type	UC Non-UC	66.6 (8/12) 29.0 (18/62)	0.0018	7.682	1.236–37.534	0.028
Donors' genetic background	Relative Non-relative	33.3 (10/30) 36.3 (16/44)	0.632	1.28	0.736–2.52	0.432
Route of administration	Enema Colonoscopy + Enema	30.7 (16/52) 45.4 (10/22)	0.036	1.832	0.899–5.212	0.126
Gender	Male Female	31.9 (15/47) 40.7 (11/27)	0.186	1.872	0.436–8.634	0.48
Donor	adult children	42.8 (9/21) 32.1 (17/53)	0.326	1.212	0.326–4.228	0.56
Number of FMT infusions	≤4 >4	60.0 (18/30) 18.1 (8/44)	0.0005	9.768	1.912–14.321	0.021

UC, ulcerative colitis; FMT, fecal microbiota transplantation.

### Follow-ups

All 74 patients were followed up for at least 3 months, 48 (64.9%) for more than 36 months, 25 (33.8%) for more than 60 months, and 10 for up to 7 years. None of the patients developed autoimmune, metabolic, or rheumatologic disorders or tumor-related diseases during the long-term follow-up. There were no deaths or serious illnesses during the follow-up. Nine children also developed rhinitis, five developed rhinitis, were underweight, and six developed constipation at different time points after FMT. None of these children had any of these diseases pre-FMT, but they might be related to the march of their diseases themselves.

**Figure 4:**
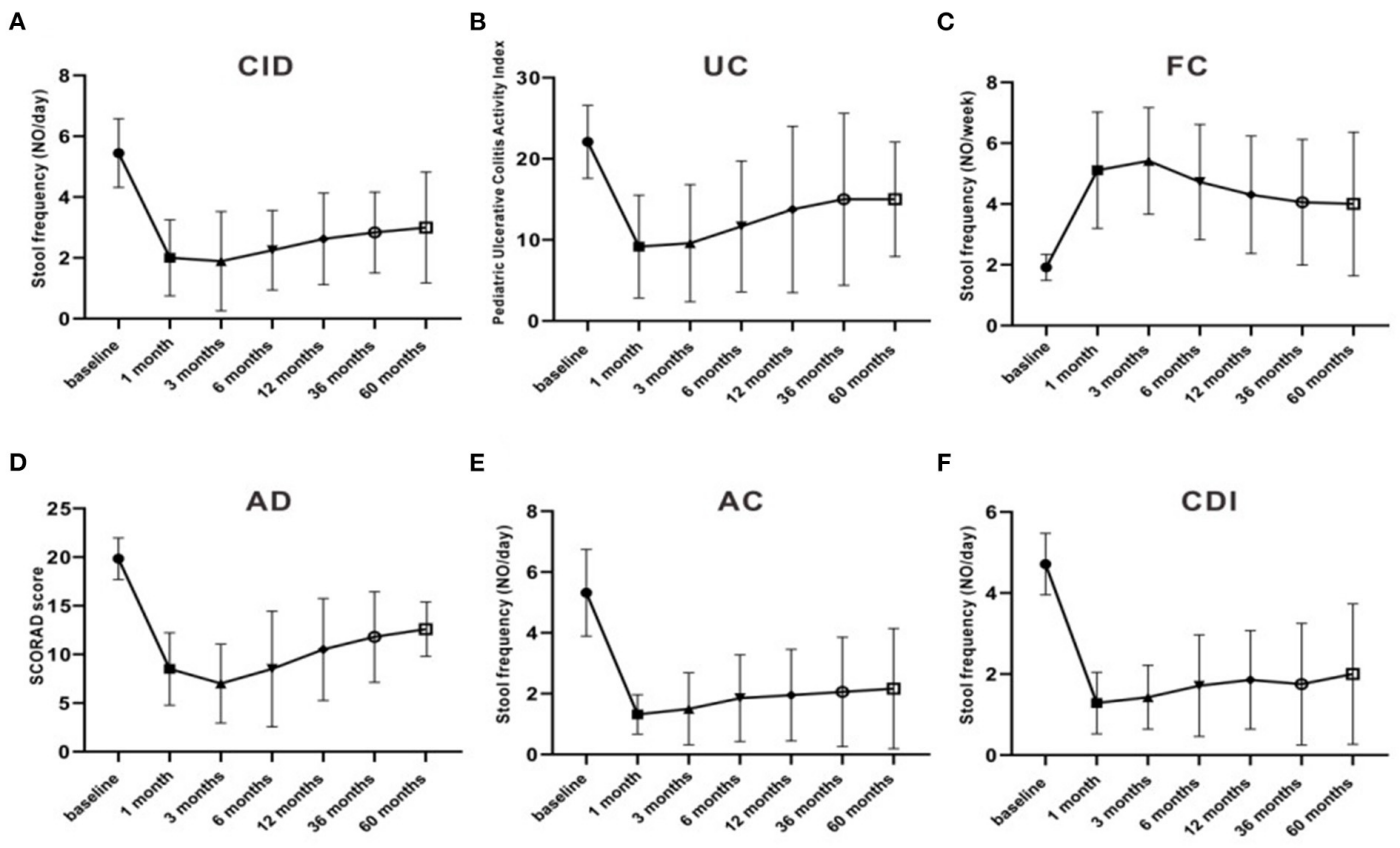
The stool frequencies and scores in different diseases and the change over time. **(A)** fecal frequency of CID; **(B)** PUCAI score; **(C)** fecal frequency of FC; **(D)** SCDAI score of AD; **(E)** fecal frequency of AC; **(F)** fecal frequency of CDI. CDI, C.difficile infection; AC, infantile allergic colitis; CID, chronic intractable diarrhea; UC, ulcerative colitis; AD, recurrent atopic dermatitis; FC, functional constipation.

Meanwhile, the clinical efficacy rates of some children gradually decreased after FMT. The PUCAI score was 22.1 ± 4.5 pre-FMT, which markedly decreased after 3 months of FMT to 9.5 ± 7.2, and then increased to 11.7 ± 6.5, 6 months after treatment. The stool frequency of CID patients dropped from 4.7 ± 0.66 times per day pre-FMT treatment to 2 ± 1.5 times per day at 1 month post-FMT treatment, then rise up to 2.5 ± 1.5 times per day at 6 months after treatment. The stool frequency of FC patients increased from 1.7± 0.66 times per week before pre-FMT treatment to 5.4 ± 1.9 times per week at 3 months after FMT treatment, then decreased to 4.3 ± 1.9 times per day at 12 months post-FMT treatment. Moreover, the stool frequency of patients with AC and CDI and the SCORAD score of patients with AD also decreased after 3 months of FMT and began to increase after 6 months (Figure 4). The clinical symptoms of these children gradually relapsed, and new medicine was added over time, especially 3–6 months after FMT. The curative effect of FMT tended to stabilize after 12 months. At 1, 3, 6, 12, 36, and 60 months after FMT, the clinical remission rates for AC were 90.9, 90.9, 80, 80, 78.6, and 83.3%, respectively; for CDI 85.7, 85.7, 71.4, 71.4, 75.0, and 66.6%, respectively; for CID 66.6, 55.5, 37.5, 37.5, 33.3, and 25.0%, respectively; for FC 66.6, 66.6, 50.0, 47.1, 40.0, and 40.0%, respectively; for UC 66.6, 66.6, 50.0, 41.6, 33.3, and 33.3%, respectively; and for AD 33.3, 50.0, 33.3, 33.3, 20.0, and 25.0%, respectively (Tables 4, 5).

**Table 4:** Clinical efficacy of patients followed up after FMT [cases *n* (%)].

**Groups**	**Followed up for 1 months**				**Followed up for 3 months**				**Followed up for 6 months**			
	**Sum(n)**	**Clinical remission**	**Clinical improvement**	**Clinical invalid**	**Sum(n)**	**Clinical remission**	**Clinical improvement**	**Clinical invalid**	**Sum(n)**	**Clinical remission**	**Clinical improvement**	**Clinical invalid**
CDI	7	6 (85.7)	0	1 (14.3)	7	6 (85.7)	0	1 (14.3)	7	5 (71.4)	0	2 (28.6)
CID	9	6 (66.6)	0	3 (33.3)	9	5 (55.5)	2 (22.2)	2 (22.2)	8	3 (37.5)	1 (12.5)	4 (50.0)
FC	18	12 (66.6)	4 (22.2)	2 (11.1)	18	12 (66.6)	3(16.6)	3 (16.6)	18	9(50.0)	4 (22.2)	5 (27.7)
UC	12	8 (66.6)	2 (16.6)	2 (16.6)	12	8 (66.6)	1 (8.3)	3 (25.0)	12	6(50.0)	2 (16.6)	4 (33.3)
AD	6	2 (33.3)	3 (50.0)	1 (16.7)	6	3 (50.0)	2 (33.3)	1 (16.7)	6	2 (33.3)	1 (16.7)	3 (50.0)
AC	22	20 (90.9)	0	2 (9.1)	22	20 (90.9)	0	2(9.1)	20	16 (80.0)	0	4 (20.0)

CDI, C. difficile infection; AC, infantile allergic colitis; CID, chronic intractable diarrhea; UC, ulcerative colitis; AD, atopic dermatitis; FC, functional constipation.

**Table 5:** Clinical efficacy of patients followed up after FMT T [cases n (%)].

**Groups**	**Followed up for 12 months**				**Followed up for 36 months**				**Followed up for 60 months**				**Follow-up lasted from 3 months to 7 years**
	**Sum(n)**	**Clinical remission**	**Clinical improvement**	**Clinical invalid**	**Sum(n)**	**Clinical remission**	**Clinical improvement**	**Clinical invalid**	**Sum(n)**	**Clinical remission**	**Clinical improvement**	**Clinical invalid**	
CDI	7	5 (71.4)	0	2 (28.6)	4	3 (75.0)	1 (25.0)	0	3	2 (66.6)	0	1 (33.3)	One patients with constipation 6 months post-FMT
CID	8	3 (37.5)	2 (25.0)	3 (37.5)	6	2 (33.3)	1 (16.6)	3 (50.0)	4	1 (25.0)	1 (25.0)	2 (50.0)	One patients with rhinitis 6 months post- FMT; one patients with constipation 1 year post- FMT; one patients with constipation 3 years post- FMT;
FC	17	8 (47.1)	2 (11.7)	7 (41.1)	10	4 (40.0)	1 (10.0)	5 (50.0)	5	2 (40.0)	0	3 (60.0)	NO
UC	12	5 (41.6)	1 (8.3)	6 (50.0)	9	3 (33.3)	2 (22.2)	4(44.4)	3	1 (33.3)	0	2 (66.6)	NO
AD	6	2 (33.3)	0	4 (66.6)	5	1 (20.0)	0	4 (80.0)	4	1 (25.0)	0	3 (75.0)	Five patients with rhinitis and two of them with underweight 3 years post-FMT
AC	20	16 (80.0)	0	4 (20.0)	14	11 (78.6)	1 (7.1)	2 (14.2)	6	5 (83.3)	0	1 (16.6)	Eight patients with rhinitis and three of them with underweight 2 years post-FMT, three patients with constipation 1 years post- FMT

CDI, C. difficile infection; AC, infantile allergic colitis; CID, chronic intractable diarrhea; UC, ulcerative colitis; AD, recurrent atopic dermatitis; FC, functional constipation; FMT, fecal microbiota transplantation.

## Discussion

Increasing evidence has demonstrated that FMT is a promising treatment method for diseases caused by flora dysregulation. However, with the growth in FMT research and applications, its safety has gradually begun to attract attention (11). There are few data regarding FMT-related AEs and long-term follow-up in children. To the best of our knowledge, this is the largest sample of children in a single center to analyze the long-term efficacy and safety of FMT. Moreover, some follow-ups reached seven years.

In our study, FMT was found to be relatively safe in children. As of January 2022, our center has performed more than 900 FMT for a variety of pediatric diseases, and no deaths have occurred. In the current study, the incidence of AE was only 13.7% (70/508), most of which were transient and mild without drug intervention. SAEs were observed four times (0.79%, 4/508) and improved after conventional drug interventions. The vast majority of AEs (88.5%) and SAE (83.3%) occurred within 48 h; thus, close surveillance of patients within 48 h of post-FMT operation is needed.

No long-term AEs have been reported during follow-up after FMT (13, 29). During our seven-year follow-up, we found that nine children developed rhinitis, five developed rhinitis, were underweight, and six developed constipation. These findings were mainly focused on AC and AD, which are allergy-related diseases (30). Allergic diseases do not exist independently. They typically progress from atopic dermatitis and food allergy in infancy to gradual march into allergic rhinitis in childhood (31). Therefore, we believe that these AEs might be related to the march of their diseases themselves rather than to post-FMT AEs. Although these diseases are not life-threatening, more controlled trials are required to identify any connections between them and FMT.

As a new therapeutic approach, FMT presented some curative advantages in the current study. The primary clinical response rate was 87.8%, which may be related to the short-term multiple FMTs performed. Our patients received almost seven FMTs on average. Numerous studies have shown that multiple FMTs are more effective than a single ones (32, 33). Meanwhile, our results showed that the AEs in children with more than four FMTs were significantly lower than those within four FMTs, indicating that children can gradually become tolerant to FMT. Considering the efficacy of FMT and AEs, we recommend performing FMT more than four times for recurrent and refractory disease.

In our study, we observed that FMT efficacy began to decline over time, especially after 3 months of FMT. Studies have also shown that after multiple FMTs, clinical remission in children with UC lasts only 126 days (34). The inability to maintain long-term efficacy is a new challenge in FMT, and exploring lasting efficacy can be a new direction in FMT studies.

Studies have shown that the decrease in donor strain populations could be examined 2–3 months post-FMT; as the donor strains decline, the clinical response of FMT will also reduce notably (35). Our study confirmed this hypothesis. In our study, all of our subjects had dysbiosis (3, 9, 10, 15, 16, 36). After FMT, the flora imbalance was significantly improved, which was accompanied by a good therapeutic effect. After 3 months, the efficacy decreased as the number of donor strains decreased. This may explain why the efficacy of FMT was stable within 3 months. For chronic diseases prone to recurrence, we considered performing FMT periodically to maintain treatment efficacy. Based on our current findings, we suggest that these children should receive more than four infusions of FMT to consolidate efficacy after 3 months of treatment.

Previous studies have pointed out that when donors are adults, more AEs and immune-related complications can occur (37). Moreover, when age-matched donors are used, fewer AEs are experienced (11). Although our current findings showed no significant difference in short-term AEs between subjects receiving age-matched donors and rigorously screened adult donors, we recommend that age-matched donors be given priority to prevent the spread of other diseases. The route of administration is thought to be another factor contributing to AEs in FMT (27), but no significant differences were found in our study. In our study, patients with UC were more likely to have short-term AEs (66.6%) and SAE (50%). This might be related to more serious intestinal mucosal injury in patients with UC (28). Therefore, for UC patients, it is best to evaluate mucosal damage by colonoscopy before FMT and wait for a slight improvement in mucosal inflammation to reduce the occurrence of AEs.

Our study has some limitations. This was not a randomized clinical trial that would have provided more important evidence. In addition, we mainly focused on the clinical safety and efficacy of pediatric subjects post-FMT but did not follow up on the variation in intestinal flora. Finally, seven years of follow-up may not be sufficient to reach conclusions on the long-term safety of FMT.

## Conclusion

Overall, FMT is a highly effective, safe, and well-tolerated treatment in children. Moreover, the clinical effects in some children gradually decrease over time. Hence, we considered the re-administration of multiple FMTs after 3–6 months to maintain efficacy.

## Data availability statement

The original contributions presented in the study are included in the article/Supplementary material, further inquiries can be directed to the corresponding author.

## Ethics statement

The studies involving human participants were reviewed and approved by Tongji Hospital, Tongji Medical College, Huazhong University of Science and Technology. Written informed consent to participate in this study was provided by the participants' legal guardian/next of kin. Written informed consent was obtained from the individual(s), and minor(s)' legal guardian/next of kin, for the publication of any potentially identifiable images or data included in this article.

## Author contributions

S-NS and Z-HH designed the research and conducted FMT. BZ performed the literature search and data extraction and drafted of the manuscript. S-XL, X-SL, and CD after FMT and follow-up. LX and M-LR collected the data. J-YH and TB performed bioinformatics analysis. All authors contributed to the article and approved the submitted version.

## Funding

This study was supported by National Clinical Key Specialty Construction Project (Pediatric Digestive Disease), No. [2011]873.

## Conflict of interest

The authors declare that the research was conducted in the absence of any commercial or financial relationships that could be construed as a potential conflict of interest. The handling editor YL is currently organizing a Research Topic with the author S-NS.

## Publisher's note

All claims expressed in this article are solely those of the authors and do not necessarily represent those of their affiliated organizations, or those of the publisher, the editors and the reviewers. Any product that may be evaluated in this article, or claim that may be made by its manufacturer, is not guaranteed or endorsed by the publisher.
