# Supplementary material for: Long-term safety and efficacy of fecal microbiota transplantation in 74 children: A single-center retrospective study
Source: Front Pediatr. 2022 Oct 11;10:964154. doi: 10.3389/fped.2022.964154 (PMC9595213; doi:10.3389/fped.2022.964154)
Supplement: Supplementary file 1 [file Data_Sheet_1.PDF]

Laboratory testing for the patients and fecal donors

Laboratory testing for donor screening
**Blood testing**
1) Blood transfusion examinations: Quantifications of hepatitis B surface antigen, hepatitis B surface antibody, hepatitis B E antigen, hepatitis B E antibody, hepatitis B core IgM antibody, hepatitis C antibody, human immunodeficiency virus antibody and treponemapallidum antibody.<br>2) TORCH examinations: Detections on toxoplasmosis IgG, toxoplasmosis IgM, rubella virus IgG, rubella virus IgM, cytomegalovirus IgG, cytomegalovirus IgM, herpes simplex virus 1/2 IgG and herpes simplex virus 1/2 IgM.<br>3) Detection on Parvovirus B19.<br>4) Epstein-barrvirus examinations: Detections on epstein-barr virus capsid antigen IgA, epstein-barr virus capsid antigen IgG, epstein-barr virus capsid antigen IgM, epstein-barr virus early antigen IgG, epstein-barr virus nuclear antigen IgG.<br>5) Blood type examination.<br>6) Lymphocyte subpopulation examination.<br>7) Food allergen examination(sIgE).<br>8) Hepatic and renal function examinations: glutamic-pyruvic transaminase, glutamic oxalacetic transaminase, total protein, albumin, globulin, prealbumin, total bilirubin, direct bilirubin, indirect bilirubin, alkaline phosphatase, gamma glutamyltranspeptidase, total cholesterol , triglycerides, high-density lipoprotein, low density lipoprotein, apolipoprotein A1, apolipoprotein B, lactic dehydrogenase, calcium, corrected calcium, phosphorus, magnesium, urea, creatinine, trioxypurine, bicarbonate radical, total bile acid, 5–nucleotidase, α-L-Fucosidase, cholinesterase, cystatin C, lipase andamylopsin.<br>9) Mycobacterium tuberculosis antibody examination (or the enzyme-linked immuno-spot assay testfor tuberculosis).<br>10) Immune system examinations: Quantifications of immune globulin A, immune globulin G, immune globulin M, alexin C3 andalexin C4.<br>11) Detection on hepatitis A-IgM.<br>12) Qualifications of C-reaction protein, erythrocyte sedimentation rate.<br>13) Multidrug-resistant bacteria .
**Stool testing**
1) Fecal routine examinations: Detections on fecal colors, character, red blood cells, white blood cells, occult blood, parasite eggs, protozoon, fat ball, rotavirus antigen and fungus.<br>2) Bacterial culture tests: Detections on Vibrio cholera, Salmonella, Shigella, Aeromonas, Plesiomonas, Pathogenic Escherichia coli, and C. difficile toxin A/B.

Other testing
1) Chest X-ray.<br>2) Urea[C^{13}] Capsule Breath Test.<br>3) Abdominal ultrasound scan.<br>4) Electrocardiography examination.

Laboratory testing for the patients

Blood testing

1) Hepatic and renal function examinations: Glutamic-pyruvic transaminas, glutamic oxalacetic transaminase, total protein, albumin, globulin, prealbumin, total bilirubin, direct bilirubin, indirect bilirubin, alkaline phosphatase, gamma glutamyltranspeptidase, total cholesterol, triglycerides, high-density lipoprotein, low density lipoprotein, apolipoprotein A1, apolipoprotein B, lactic dehydrogenase, calcium, Correction of calcium, phosphorus, magnesium, urea, creatinine, trioxypurine, bicarbonate radical, total bile acid, 5–nucleotidase, α-L-Fucosidase, cholinesterase, cystatin C, lipase and amylopsin.

2) Food allergen examination (sIgE).

3) Lymphocyte subpopulation examination.

4) Detection on hepatitis A-IgM.

5) Blood transfusion examinations: Quantifications of hepatitis B surface antigen, hepatitis B surface antibody, hepatitis B E antigen, hepatitis B E antibody, hepatitis B core IgM antibody, hepatitis C antibody, human immunodeficiency virus antibody and treponemapallidum antibody.

6) TORCH examinations: Detections on toxoplasmosis IgG, toxoplasmosis IgM, rubella virus IgG, rubella virus IgM, cytomegalovirus IgG, cytomegalovirus IgM, herpes simplex virus 1/2 IgG and herpes simplex virus 1/2 IgM.

7) Detection on Parvovirus B19.

8) Blood coagulation examinations: Detections on prothrombin time, prothrombin activity, international normalized ratio, fibrinogen, activated partial thromboplastin time, thrombin time and d-dimer.

9) Mycobacterium tuberculosis antibody examination (or the enzyme-linked immuno-spot assay testfor tuberculosis).

Stool testing

1) Fecal routine examinations: Detections on fecal colors, character, red blood cells, white blood cells, occult blood, parasite eggs, protozoon, fat ball, rotavirus antigen and fungus.

2) Bacterial culture tests: Detections on *Vibrio cholera*, *Salmonella*, *Shigella*, *Aeromonas*, *Plesiomonas*, Pathogenic Escherichia coli, and C. difficile toxin A/B.

Other testing

1) Abdominal ultrasound scan(intestinal adhesion)

2) Electrocardiography examination.
